# *In Vitro* Evaluation of Essential Oils Derived from* Piper nigrum* (Piperaceae) and* Citrus limonum* (Rutaceae) against the Tick* Rhipicephalus (Boophilus) microplus* (Acari: Ixodidae)

**DOI:** 10.1155/2017/5342947

**Published:** 2017-10-08

**Authors:** Rafaelle Vinturelle, Camila Mattos, Jéssica Meloni, Jeane Nogueira, Maria Júlia Nunes, Itabajara S. Vaz, Leandro Rocha, Viviane Lione, Helena C. Castro, Evelize Folly das Chagas

**Affiliations:** ^1^Laboratório de Estudos em Pragas e Parasitos, Universidade Federal Fluminense, IB, Departamento de Biologia Celular e Molecular–GCM, Niterói, RJ, Brazil; ^2^Programa de Pós-Graduação em Ciências e Biotecnologia, Universidade Federal Fluminense, Niterói, RJ, Brazil; ^3^Laboratório de Tecnologia de Produtos Naturais, Faculdade de Farmácia, Universidade Federal Fluminense, Niterói, RJ, Brazil; ^4^Programa de Pós-Graduação em Ciências Aplicadas a Produtos para Saúde, Departamento de Farmácia e Administração Farmacêutica, Faculdade de Farmácia, Universidade Federal Fluminense, Rua Doutor Mário Viana 523, 24241-000 Niterói, RJ, Brazil; ^5^Bellarome Aromoterapia, Centro, Rio de Janeiro, RJ, Brazil; ^6^Centro Universitário Augusto Motta (UNISUAM), Rio de Janeiro, RJ, Brazil; ^7^Centro de Biotecnologia e Faculdade de Veterinária, Universidade de Federal do Rio Grande do Sul, Porto Alegre, RS, Brazil; ^8^Instituto Nacional de Ciências e Tecnologia em Entomologia Molecular (INCT-EM), Rio de Janeiro, RJ, Brazil; ^9^Faculdade de Farmácia, Universidade Federal do Rio de Janeiro, Rio de Janeiro, RJ, Brazil

## Abstract

The present research aimed to study the chemical composition and acaricidal activity of* Citrus limonum* and* Piper nigrum* essential oils against the cattle tick* Rhipicephalus microplus*. GC-MS analysis of* C. limonum* essential oil showed limonene (50.3%), *β*-pinene (14.4%), and *γ*-terpinene (11.7%) as the major components;* P. nigrum* oil was mainly composed of *β*-caryophyllene (26.2%), *σ*-ocymene (5.8%), and *α*-pinene (5.5%). Acaricide activity was evaluated at concentrations of 2.5, 5.0, and 10.0% (v/v) of each plant oil, as well as 1 : 1 combination of both oils (5% : 5%, 2.5% : 2.5%, and 1.25% : 1.25% each), by immersing engorged* R. microplus* females for one minute. The LC90 of oils from* C. limonum, P. nigrum,* and the combination were 4.9%, 14.8%, and 5.1%, respectively.* C. limonum* essential oil caused 100% mortality of engorged females at the highest concentration (10%).* P. nigrum* essential oil inhibited egg-laying by up to 96% in a concentration-dependent manner, suggesting it reduces tick fecundity. When combined, the oils presented toxicity as to* C. limonum* oil alone, but with stronger inhibition of oviposition (5% : 5%), indicating a possible additive effect against* R. microplus*. The present data provide support for further investigation of novel natural products to control bovine tick infestations.

## 1. Introduction

The tick* Rhipicephalus (Boophilus) microplus* (Canestrini, 1887) is an ectoparasite of cattle [1], present in tropical and subtropical areas of America, Africa, Asia, and Australia in latitudes between 32°N and 32°S [2, 3]. About the damage caused by this parasite, direct losses in the production of milk and meat, leather damage caused by inflammatory reactions at the sites of tick attachment, appearance of myiasis, and the transmission of diseases such as bovine tick fever (caused by protozoa of the genus* Babesia* and by bacteria of the genus* Anaplasma*) could be mentioned [4]. The economic losses of Brazil related to ticks are estimated by 3.24 billion dollars a year, represented by the direct action of the parasite in the animals and by the cost of the control systems [5].

One of the strategies to minimize the economic losses caused by this pest involves reducing tick populations to economically acceptable levels [6]. But the inherent problems in controlling this parasite are acaricide resistance, and environmental residues resulted in only partial success of tick control [7]. The searches for environmentally safe products have accelerated the research on botanical acaricides [8, 9]. Plants have been used as food, spices, and medicines for thousands of years. These medicines initially took the form of crude drugs such as a variety of plants formulations. Natural products have been the origin of many important molecules in drug discoveries. Different culinary herbs have been screened for their biological activities. The active ingredients from plants are known to possess insecticidal, growth inhibiting, antimoulting, and repellent activities [8]. Thus, there is increasing interest in alternative methods for tick control and growing demand for nonchemical products in livestock that are safer to animals and the environment [10, 11]. Botanical alternatives, such as essential oils, are currently receiving particular attention [12].

In Brazil, the Rutaceae family comprises about 38 genera and 22 species [13].* Citrus limonum* (L.) comprises 3 species and various hybrids. Species of this genus are known to show monoterpenes and sesquiterpenes, such as limonene and *α*-pinene [14, 15]. Essential oils of this kind have been evaluated for presenting biological activities such as insecticide, cytotoxic, and antibacterial activities [16].

*Piper nigrum* (L.), popularly known as black pepper, is a member of family Piperaceae [17, 18]. The genus* Piper* includes more than 1000 species, but the best known are* P. nigrum, Piper longum,* and* Piper betle* [19, 20]. The fruits of* P. nigrum* contain piperine, a compound that has already shown leishmanicidal activity [21]. Furthermore, secondary metabolites of this species possess active compounds with insecticidal activity, antibacterial, antifungal, and others [20, 22].

The aim of this work is to develop natural products, like essential oils, for the combat of the cattle tick.

## 2. Materials and Methods

### 2.1. Essential Oil

*Citrus limonum* and* Piper nigrum* essential oils were obtained commercially in Bellarome, RJ, Brazil commercial Ltda. According to the company,* C. limonum* essential oil was obtained from the juice industry peel residue, which was pressed and the oil was washed out with water of the material, while the dried fruit of* P. nigrum* was steam distilled for 60 minutes to obtain the essential oil.

### 2.2. Analysis by GC/MS

The crude essential oil was analyzed on a GC-MS QP2010 (SHIMADZU) gas chromatograph equipped with a mass spectrometer using electron impact ionization detection. The gas chromatographic (GC) conditions were as follows: injector temperature: 260°C; detector temperature: 290°C; carrier gas (Helium): flow rate 1 mL/min; and split injection with split ratio 1 : 40. Oven temperature was raised from 60°C to 290°C at a rate of 3°C/min. One microliter of each sample, dissolved in CH2Cl2 (1 : 100 mg/*μ*L), was injected on a RTX-5 column (i.d. = 0.25 mm, length 30 m, film thickness = 0.25 *μ*m). Mass spectra (MS) were recorded 70 eV and scan rate of 1 scan/s. The retention indices were calculated by interpolation of retention times of the substances to the retention times of a mixture of aliphatic hydrocarbons (C7-C40) (Sigma-Aldrich Corporation, St Louis, MO) analyzed in the same conditions (Van den Dool H. & Kratz). The identification of substances was performed by comparison of their retention indices and mass spectra with those reported in literature [23]. The MS fragmentation pattern of compounds was also checked with NIST mass spectra libraries.

### 2.3. Preparation of the Samples for Acaricidal Test

The experiment was conducted in two phases. Essential oils were tested separately and after mixing were performed at equal concentrations, in 1 : 1 proportions (50%* Citrus limonum *and 50%* Piper nigrum*). The essential oils of* C. limonum* and* P. nigrum* were dissolved in an aqueous solution with 2% dimethyl sulfoxide (v/v) as an emulsifying agent, as described by Gazim et al. in [24]. Solutions were serially diluted in order to obtain the concentrations of 2.5%, 5%, and 10% (v/v), obtaining a final volume of 5 mL. The emulsifying solution (2% DMSO) was used as the negative control. A control with water was also carried out. Amitraz (2 *μ*L/mL) and Deltamethrin (1 *μ*L/mL) in water were used as positive control. The test was repeated three times in duplicate.

### 2.4. *Rhipicephalus microplus* Ticks for Bioassays

The engorged females ticks of* R. (B.) microplus* were collected from infested animals from a farm of Rio Grande do Sul (Brazil) without history of acaricide use. The tick population was maintained based on artificial infestations on calves at Faculdade de Veterinária from Federal University of Rio Grande do Sul (UFRS), Brazil. All experiments were conducted following the guidelines of the Ethics Committee on Animal Experimentation of UFRS and FEPAGRO. The ticks were thoroughly washed with tap water, dried on filter paper towel, and used in the adult immersion test.

### 2.5. Adult Immersion Test (AIT)

The AIT was performed as described by Drummond et al. [25] with minor modifications. The ticks were distributed to groups randomly (20 engorged females per group). The different groups of* R. (B.) microplus* were immersed for 1 min in 5 mL of the respective concentrations (2.5%, 5%, and 10%) of* C. limonum *and* P. nigrum* by placing them directly into a 50 mL Falcon-type tube which was gently agitated at room temperature. In experiments of additive effects we used the ratio 1 : 1 (vol/vol) of each oil, with a final concentration of 2.5%, 5%, and 10%. Ticks were recovered from the solutions and dried and each group was transferred to Petri dish (9 cm diameter, 1,5 cm high), weighed, and kept in BOD incubator at a temperature of 28°C and relative humidity of 70–80% for oviposition. This experiment was performed three times in duplicate. The emulsifying solution (2% DMSO) was used as the negative control, while Amitraz (2 *μ*L/mL) and Deltamethrin (1 *μ*L/mL) in water were used as positive control. The mortality was observed during 15 days. The dead ticks were diagnosed based on three specific signs and features: increasing cuticle darkness and stopping Malpighian tube movement and hemorrhagic skin lesions. After 15 days, the eggs laid were placed in glass tube, weighed, and observed separately at the same condition of incubation for the next 30 days for visual estimation of hatching rate.

The percentage inhibition of oviposition was calculated as follows.

Reproductive index (RI) = average weight of eggs laid (mg)/average weight of females before treatment (g).

Percentage inhibition of oviposition (IO%) = RI of control group − RI of treated group/RI control group × 100.

### 2.6. Statistical Analyses

Data were expressed as the mean ± standard error of the mean (SEM). Groups were compared using ANOVA one-way test. A *p* value of less than 0.05 was considered significant. Statistical analysis was performed using GraphPad Prism 6.0 (GraphPad Software Inc., San Diego, USA) software.

The efficacy was assessed by measuring female mortality (%) and the lethal concentrations for 50% (LC50) and 90% (LC90) with their 95% confidence limits (CL) values were estimated by applying regression equation analysis to the probit transformed data of mortality.

## 3. Results

### 3.1. Identification of the Components by GC/MS

The composition of the oil from* Citrus limonum* and* Piper nigrum*, together with the relative retention index and percentage of the identified compounds, is listed in Table 1. A total of 29 compounds representing 96.3% of the total oil were identified from* C. limonum* essential oil. The chromatographic profile of* C. limonum* showed a complex mixture of monoterpenes (90.3%) and sesquiterpenes (3.1%).* C. limonum* oil was composed mainly by the monoterpenes limonene (50.3%), *β*-pinene (14.4%), and *γ*-terpinene (11.7%).

In* P. nigrum* essential oil, 42 compounds were identified, comprising 89% of the oil. The essential oil was characterized by the presence of sesquiterpenes (58.9%) and monoterpenes (26.3%). The sesquiterpene *β*-caryophyllene was identified as the major compound, representing 26.2% of the oil, followed by the monoterpenes hydrocarbons *σ*-ocymene (5.8%) and *α*-pinene (5.5%).

### 3.2. *Rhipicephalus* (*Boophilus*)* microplus* Engorged Female Mortality Rate

**Figure 1:**
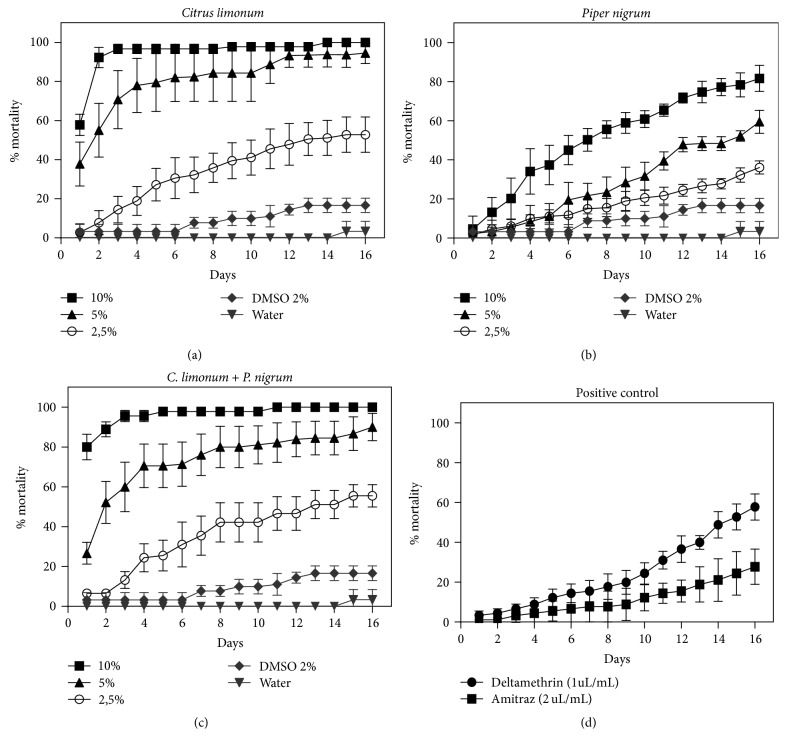
Mortality percentage of* R. microplus* engorged females exposed to* Citrus limonum* and* Piper nigrum* essential oil. Adult Immersion Test (AIT) on mortality of the tick* R. microplus*. Groups treated with different concentrations of essential oil of* C. limonum* (a),* P. nigrum* (b), the combination of both oils (c), and positive control were the commercial acaricides Deltamethrin and Amitraz. In combination oils used the 1 : 1 (vol/vol) concentration of each oil. Results are means ± SD of five experiments.

Laboratory tests were carried out to determine the toxicity of the essential oils from* C. limonum* and* P. nigrum* on engorged females of* R*.* (B.) microplus*. The results referents to mortality rate of engorged females after adult immersion test (AIT) are present in Figure 1 and Table 2. Essential oil from* C. limonum* exhibited 90% of mortality on engorged females in the highest concentration tested after 48 h, reaching 100% at the end of the experiments (day 16). At concentration of 5%,* C. limonum* achieved approximately 80% mortality rate in the fourth day and getting the most for 90% mortality at the 12th day. The 2.5%* C. limonum* essential oil led to 50% mortality rate on day 16 after treatment (Figure 1(a)).

The essential oil of* P. nigrum* also demonstrated high efficacy against* R. (B.) microplus* tick using the highest concentration (10%), which caused a mortality of 80% of females on the 16th day. The concentrations of 5 and 2.5% essential oil led to a mortality rate of 60% and 35%, respectively (Figure 1(b))

The toxic effect of the mixture of essential oils was also studied (Figure 1(c)). The mixture of* C. limonum* and* P. nigrum* oil induced 100% death at a concentration of 10% at the end of treatments. The results of the groups treated with the combination of the oils, at all concentrations tested, were very similar to the results of the* C. limonum* oil. These results demonstrate the dose-dependent character of* R. (B.) microplus* mortality and there was a significant difference between all treatments when compared with control group.

When AIT was performed with positive controls, the mortality of engorged females of* R. (B.) microplus* for Deltamethrin and Amitraz was 57.7% and 27.7%, showing a level of resistance status (Figure 1(d))

Table 2 summarizes LC50 and LC90 values for individuals and combined treatments of* C. limonum* and* P. nigrum* oils against engorged females. The most potent modified oil was* C. limonum* with a LC90 of 4.9% whereas the combined oils exhibited a LC90 of 5.1%.

### 3.3. Effect of Essential Oil of* Citrus limonum*,* Piper nigrum,* and Their Mixture on* Rhipicephalus* (*Boophilus*)* microplus* Female Oviposition and Hatching

In the AIT the efficacy of treatment against engorged females was also evaluated by measuring egg production. The results are presented in Table 3. Ours results showed that* C. limonum* and the oil mixture have the best inhibition of oviposition rate, which is 100% in a dilution of 10%. However, at 5% the group with combined oils exhibited greater inhibition of oviposition with 94%, compared to 81.6% inhibition by individual* C. limonum*.* P. nigrum* essential oil, at concentrations of 10%, 5%, and 2.5%, inhibited egg-laying by 96%, 83%, and 50%, respectively. Data were significantly different in relation to the control (DMSO 2% and water) group (*p* < 0.001). The few eggs laid by the females treated with 5% of* C. limonum* essential oil have not hatched into larvae. This result showed that oviposition is also dose-dependent (Table 3). Thus, analysis of variance *p* < 0.05 for treatments confirmed the positive development of inhibition oviposition with increasing concentration. The mixture is effective against* R. (B.) microplus* engorged females since its effect is fast and is expressed at a low concentration on reducing the rate of lay. However, the essential oils did not interfere in the hatchability of the larvae.

## 4. Discussion

The analysis of chemical composition of* Citrus limonum* essential oil by GC/MS showed as major compounds limonene (50.3%), *β*-pinene (14.4%), and *γ*-terpinene (11.7%). This result agrees with that found by others groups in previous studies with species of genus* Citrus*, where the same chemical compounds can be found, although in different proportions [26]. The essential oil chemical compositions of* Piper nigrum* consisted primarily of the sesquiterpene *β*-caryophyllene and the monoterpenes hydrocarbons *σ*-ocymene and *α*-pinene, at 26.2%, 5.8%, and 5.5%, respectively.

Essential oil from* C. limonum* exhibited greater activity on engorged females than* P. nigrum*, reaching a mortality of 100% at concentrations of 10%, on 16th day. The values of LC50 and LC90 of* C. limonum* were 2.2% and 4,9%, respectively.* P. nigrum* exhibited LC50 of 3.7% and LC90 of 14.8%.

Components such as limonene and pinene have already been reported to possess acaricidal activity against ticks. Limonene, extracted from* Lippia alba* (Verbenaceae) essential oils of two chemotypes, demonstrated toxic effect on* R. (B.) microplus* larvae [27]. In another study, it was shown that limonene inhibited 91% of* R. (B*.*) microplus* hatching eggs at concentration of 1.25 *μ*g/mL [28]. Moreover, this chemical constituent has also exhibited insecticide potential and proved to be highly toxic to mosquito larvae of* Aedes aegypti* (Diptera: Culicidae),* Culex pipiens pallens* (Diptera: Culicidae), and* Ochlerotatus togoi* (Diptera: Culicidae) [29], for grain insect pests stored* Rhyzopertha dominica* (Coleoptera: Bostrichidae) and* Tribolium castaneum* (Coleoptera: Tenebrionidae) [30] and lice* Pediculus humanus capitis* (Anoplura: Pediculidae) [31]. Acaricide activity in* R. (B.) microplus* larvae has also been demonstrated with essential oil of* Cunila incana* (Lamiaceae), rich in *β*-pinene (27.5%) and *α*-pinene (26.7%), reaching 100% mortality with concentration of 2.5 *μ*L/mL [32]. This suggests the major constituents of the essential oil are acting in an additive or added effect manner, thus contributing equally to the observed toxic effects. However, the added effect action of other minor constituents cannot be disregarded. Some studies have shown that essential oils have higher efficiency than their isolated components [33, 34].

Chungsamarnyart and Jansawan in [35] observed that the essential oil of* Citrus maxima* and* Citrus sinensis* showed over 90% mortality of* R. (B.) microplus* engorged females at a concentration of 1/5 in 24 hours. This concentration is higher than that found in our study. Already* Citrus reticulata* oil demonstrated a similar profile to* C. limonum*, reaching about 95% mortality within 24 h at a concentration of 1/10. Plants of the same genus could have the same class of substances and so present similar activity. However, such substances may vary in concentration according to seasonality, circadian rhythm, and development of the plant [36].

*Citrus limonum* has already shown biological activity in several species of insects. The essential oil of* C. limonum* caused antifeedant activity in* Spodoptera litura* larvae (Lepidoptera: Noctuidae) and took the mortality* Spodoptera littoralis* larvae (Lepidoptera: Noctuidae) in the test by contact [37, 38]. Pavela in [39] also demonstrated that essential oil of these specie showed fumigant and contact activity on the house fly* Musca domestica* (Diptera: Muscidae).

The reduction in the number of* C. limonum* eggs is probably due to female mortality in the first few days after treatment. However, it is possible that the essential oil of* P. nigrum* has caused a reduction in female's fecundity, since exposure to sublethal concentrations of the oil significantly reduced the number of eggs. The same effect was observed with the essential oil of* Piper marginatum* used as fumigant against the mite* Tetranychus urticae* (Acari: Tetranychidae), where the fecundity was drastically reduced. The *β*-caryophyllene was one of the main compounds (16%) found in the essential oil of* P. marginatum*, as well as the oil of* P. nigrum* in the present work. This constituent was also evaluated against* T. urticae* and this also reduced the females' fecundity in sublethal concentrations [40].

According to chemical analysis, the essential oil of* P. nigrum* majority has the sesquiterpene *β*-caryophyllene (26.2%) and the monoterpenes hydrocarbons *σ*-ocymene (5.8%) and *α*-pinene (5.5%). However, Ferreira et al. in [41] analyzed the essential oil from* P. nigrum* and found as main components the sesquiterpene *β*-caryophyllene (21.8%) and the monoterpenes limonene (19.8%) and 3-*σ*-carene (14.3%). The constituents of essential oils can vary with environmental conditions, such as climate, soil type and brightness. The biological properties of *β*-caryophyllene have been confirmed in previous studies involving* Leptinotarsa decemlineata *(Coleoptera: Chrysomelidae),* S. littoralis* [42], larvae of* A. aegypti* [43], and* T. urticae* [44]. The compound *α*-pinene has been reported with insecticidal activity in larvae of* C. pipiens* [45] and as a fumigant against the adult mushroom fly* Lycoriella mali* (Diptera: Sciaridae) [46].

Plants of the genus* Piper* have demonstrated toxic effect on* R. (B.) microplus.* Ferraz et al. in [47] showed that the essential oils of* Piper mikanianum* and* Piper xylosteoides* were active against* R. (B.) microplus* larvae (LC50 of 2.3 and 6.2 *μ*L/mL, resp.).* Piper tuberculatum* and* Piper aduncum* extracts were also highly effective against the larvae and engorged female tick* R. (B.) microplus* [48, 49]. In addition, other studies have shown that the essential oil of* P. nigrum* has insecticidal activity against mosquitoes* A. aegypti*,* A. stephensi* (Diptera: Culicidae), and* C. quinquefasciatus* (Diptera: Culicidae) [50].

Ours results showed that the oil mixture has a high acaricidal activity on engorged females of* R. (B.) microplus*, reaching 100% mortality, at a concentration a little larger than that of the* C. limonum* essential oil (LC90 = 4,9%). However, the oils combination showed to be more effective in the inhibition of oviposition compared to the oil* C. limonum*. Considering that* C. limonum* oil caused high mortality of females and* P. nigrum* oil reduced fertility, it is possible that the essential oils have an additive effect when mixed in the same solution, exhibiting both effects against* R (B.) microplus* tick females. Yessinou et al. in [51] recently showed that the mixture of* Syzygium aromaticum* (Myrtaceae) and* Cymbopogon citratus* (Poaceae) essential oils was more effective in engorged females of* R. (B.) microplus* than when tested separately, confirming the action potential of essential oils when used simultaneously.

## 5. Conclusion

This study demonstrates that the essential oil of lemon has a high acaricide activity against the tick* R. (B.) microplus*, especially in its engorged female and egg stages, cutting its cycle. Thus, it can be a possible candidate for biocontrol against this cattle tick.

## Figures and Tables

**Table 1:** Percentage composition of *Citrus limonum* and *Piper nigrum* oils.

Constituents	RI	*Citrus limonum* (%)	*Piper nigrum* (%)
*Monoterpenes hydrocarbons*			
*α*-Thujene	927	0.6	1.0
*α*-Pinene	935	2.6	5.5
Camphene	950	0.1	—
Sabinene	975	2.8	0.9
*β*-Pinene	978	14.4	4.1
Myrcene	992	2.0	0.3
Phellandrene	1007	—	4.1
*δ*-3-Carene	1013	—	0.9
*α*-Terpinene	1018	0.2	0.7
*σ* -Ocymene	1026	2.9	5.8
Limonene	1030	50.3	2.1
*β*-E-Ocymene	1037	0.1	—
*β*-Z-Ocymene	1048	0.1	—
*γ* - Terpinene	1060	11.8	0.5
Terpinolene	1086	0.1	—
p-Mentha-2,4(8)-Diene	1091	—	0.3
*Oxygenated monoterpenes*			
1,8-Cineol	1034	0.1	—
Linalool	1101	0.2	—
Limonene oxide –cis	1135	0.1	—
Limonene oxide –trans	1139	0.1	—
Carvenon	1147	0.1	—
Terpinen-4-ol	1178	0.1	—
*α*-Terpineol	1191	0.4	0.1
Nerol	1224	0.1	—
Neral	1242	1.1	—
*Sesquiterpene hydrocarbons*			
*δ*-Elemene	1340	—	0.9
*α*-Cubebene	1353	—	0.5
*α*-Copaene	1379	—	4.2
*β*-Cubebene	1383	—	0.2
*β*-Elemene	1395	—	1.1
Cyperene	1404	—	0.2
Bergamotene	1418	0.04	—
*β*-Caryophyllene	1426	—	26.2
*β*-Copaene	1429	—	0.2
Trans-Bergamotene	1439	—	3.9
*α*-Germanene	1443	—	0.5
(z)-*β*-Farnese	1446	—	0.2
*α* -Humulene	1458	—	2.9
9- EPI - (E)-Caryophyllene	1466	—	0.3
*γ*-Muurolene	1481	—	0.9
Trans-Muurola-4(14),5-Diene	1485	—	1.5
*β*–Selinene	1491	—	1.1
*α* –Selinene	1499	—	1.1
Bisabolene	1506	0.1	—
*β*-Bisabolene	1512	1.8	4.1
*γ*-Cadinene	1519	—	0.6
*δ*-Cadinene	1528	—	2.1
Germacrene B	1562	—	0.5
*Oxygenated sesquiterpenes*			
Bergamotol *α*-trans	1439	1.1	—
Caryophyllene oxide	1589	—	4.2
Junenol	1624	—	0.6
Epi-*α*-Muurolol	1648	—	0.4
*α*-Muurolol	1652	—	0.3
*α*–Cadinol	1660	—	0.4
*Others*			
Neril format	1272	1.9	—
Neril acetate	1367	0.9	—
Geranyl acetate	1386	0.3	—
Guaiol acetate	1729	—	0.7
Benzil benzoato	1771	—	3.0
Manool	2063	—	0.2
*Total identified*		96.3	89.0

RI: retention index on DB-5MS column in reference to *n*-alkanes.

**Table 2:** LC50 and LC90 (%) obtained for the engorged female of the essential oils of *Citrus limonum*, *Piper nigrum,* and their combination on *R. microplus*.

Essential oil	LC50 (%)	LC90 (%)
*Citrus limonum*	2.2	4.9
*Piper nigrum*	3.7	14.8
*C. limonum *+* P. nigrum*	2.2	5.1

**Table 3:** Mortality percentage (MAM) of *R. microplus* engorged females, exposed to different concentrations of *Citrus limonum* and *Piper nigrum*, and effect reproductive index (RI), inhibition of oviposition (IO), and larvae hatching (LH).

Concentration (%)	MAM (%) ± SE	RI ± SE	IO (%)	LH
*Citrus limonum*				
10%	100.0 ± 0.0^a,b^	0.0 ± 0.0^a,b^	100	—
5%	94.4 ± 5.7^a,b^	0.1 ± 0.1^a,b^	81.6	Without hatching
2.5%	52.8 ± 12.8^a,b^	0.3 ± 0.1^a,b^	35	There was hatching
*Piper nigrum*				
10%	81.7 ± 6.7^a,b^	0.0 ± 0.0^a,b^	96	Without hatching
5%	59.4 ± 9.4^a,b^	0.1 ± 0.0^a,b^	83	There was hatching
2.5%	36.1 ± 3.9^a^	0.2 ± 0.0^a,b^	50	There was hatching
*C. limonum + P. nigrum*				
10%	100.0 ± 0.0^a,b^	0.0 ± 0.0^a,b^	100	—
5%	90.0 ± 10.0^a,b^	0.0 ± 0.0^a,b^	94	Without hatching
2.5%	55.6 ± 9.0^a,b^	0.1 ± 0.0^a,b^	67	There was hatching
Amitraz (2 *µ*L/mL)	27.8 ± 7.9^a^	0.0^a,b^	100	—
Deltamethrin (1 *µ*L/mL)	57.8 ± 9.0^a,b^	0.0^a,b^	100	—
DMSO 2%	16.7 ± 3.4	0.5 ± 0.0	0	There was hatching
Water	3.3 ± 3.3	0.5 ± 0.0	0	There was hatching

MAM: mean % adult mortality within 16 days, SE: standard error, RI: reproductive index, IO: (%) percent inhibition of oviposition, and LH: Larvae hatching after females treatment. ^a^Significant difference in relation to the negative control (water). ^b^Significant difference in relation to the negative control (2% DMSO) (ANOVA one way *p* = <0.0001).
